# Nonlinear relationship between early postnatal weight gain velocity and neurodevelopmental outcomes in very-low birth weight preterm infants: A secondary analysis based on a published prospective cohort study

**DOI:** 10.3389/fped.2022.944067

**Published:** 2022-11-08

**Authors:** Zhongchen Luo, Beibei You, You Zhang, Jiao Tang, Zehong Zheng, Yuling Jia, Li Wang, Dan Zeng, Hong Li, Xiuhong Wang

**Affiliations:** ^1^ School of Nursing, Guizhou Medical University, Guiyang, China; ^2^ School of Foreign Languages, Chongqing Medical University, Chongqing, China; ^3^ School of Nursing, Chongqing Medical University, Chongqing, China; ^4^ Department of Nursing, The First Affiliated Hospital of Chongqing Medical University, Chongqing, China; ^5^ Engineering Training Center, Guizhou Minzu University, Huaxi, Guiyang, China; ^6^ Department of Nursing, The Affiliated Hospital of Guizhou Medical University, Guiyang, China

**Keywords:** neurodevelopmental outcomes, weight gain velocity, very low birth weight premature infants, early postnatal, prospective cohort study

## Abstract

**Background:**

Extrauterine growth restriction among the very-low birth weight preterm infants (VLBWPIs) is associated with poorer cognitive development outcome, while the rapid weight gain in infancy increases the long-term risk of obesity and noncommunicable disease among VLBWPIs. However, the results of research on the association between early postnatal growth velocity and neurodevelopmental outcomes in VLBWPIs are still limited and controversial.

**Objective:**

We aimed to explore the association between the growth velocity in early postnatal and neurodevelopmental impairment (NDI) among VLBWPIs.

**Methods:**

This study was a secondary analysis of a previously published prospective cohort. It was based on data on 1,791 premature infants with a birth weight of less than 1500 g, registered in the database of the Premature Baby Foundation of Taiwan between 2007 and 2011. A binary logistic regression model was used to evaluate the association between the weight gain velocity in different periods [from birth to 6 months corrected age (CA), 6 to 12 months CA, and 12 to 24 months CA] and NDI, respectively. The generalized additive model and the smooth curve fitting (penalized spline method) were used to address nonlinearity, and a two-piece-wise binary logistic regression model was added to explain the nonlinearity further.

**Results:**

Nonlinearities were observed between NDI and the weight gain velocity from birth to 6 months CA [inflection point 20.36, <inflection point: odds ratio (OR) = 0.75, 95% confidence interval (CI) 0.67–0.84, >inflection point: OR = 1.01, 95% CI 0.97–1.05], 6–12 months CA [inflection point 9.44, <inflection point: OR = 0.89, 95% CI 0.84–0.94, >inflection point: OR = 1.05, 95% CI 1.05–(1.00, 1.11)], and 12–24 months CA [inflection point 16.00, <inflection point: OR = 0.93, 95% CI 0.88–0.98, >inflection point: OR = 1.75, 95% CI 1.05–(0.96, 3.08)].

**Conclusion:**

The neurodevelopmental benefits from a rapid weight gain velocity from birth to 24 months CA might be limited once the growth pace reaches an optimum level. It would help find a pattern of growth that facilitates optimal neurodevelopment, yet minimizes negative health consequences associated with overnutrition further.

## Introduction

Advanced neonatal care has dramatically decreased the mortality of very-low birth weight preterm infants (VLBWPIs, with a birth weight ≤1500 g and gestational age at birth <37 weeks) in recent decades (1–3), and the rate of VLBWPIs was estimated to account for 0.8% of live births in Taiwan (about 200,000 infants in total born each year) (1). The weight gain plays a significant role in neurodevelopment among VLBWPIs. Extrauterine growth restriction among VLBWPIs is associated with poorer cognitive development outcome (4–6). However, the correlation between the early weight gain velocity after birth and neurodevelopmental impairment (NDI) in preterm infants remains controversial. A large number or studies reported that there is a positive association between postnatal weight growth with neurocognitive outcomes in different periods among low-weight preterm infants (7, 8). However, other articles revealed a nonlinear association between them. Pylipow et al. (9) revealed a nonlinear association between the postnatal growth velocity in the first four postnatal months of infants with intrauterine growth restriction (<2211 g at ≥37 weeks’ gestation) and later cognitive function. Infants weight gain ranged from 1,059 to 5,119 g had lower achieved cognitive testing scores apparent at both extremes (an inverted *J*-shape), with both extremes associated with negative effects. Kim et al. (10) examined the growth velocity at different time periods after birth to school age in relation to neurocognitive outcomes among small for gestational age preterm infants, and only postnatal weight gain in the neonatal intensive care unit (NICU) was positively correlated with good neurodevelopmental outcomes. Additionally, Meyers et al. (11) reported that linear growth-restricted infants born <29 weeks with weight gain out of proportion to linear growth was associated with poorer 2-year neurodevelopmental outcomes, and infants with high body mass index (BMI) were more likely to have neurodevelopmental impairment compared with those with low-to-normal BMI. These inconsistencies may be attributable to the disparity in population, design, adjustment for covariates, especially the timing of measurement, different outcome indicators of neurodevelopmental impairment, or calculation of the weight gain velocity.

In addition, the rapid early postnatal weight growth pace among VLBWPIs is positively associated with a higher risk of overweight/obesity and another noncommunicable disease. The rapid weight gain in infancy increases the long-term risk of obesity and noncommunicable disease among low-weight preterm infants including insulin resistance and metabolic syndrome, cardiovascular risk, adiposity, higher blood pressure, insulin resistance, dyslipidemia, and endothelial dysfunction (12, 13). It is noteworthy that in line with advances in medical and nutritional care during the past decade, the weight growth pace of preterm infants in the postnatal period is rapid and the rate of short-term extrauterine growth restriction is reduced (14, 15). Overemphasizing the prevention of failure to thrive postnatally and ignoring the effect of rapid acceleration of growth on metabolic syndrome later in life may be also a harmful pattern of growth. Achieving a pattern of growth that facilitates optimal neurodevelopment, yet minimizes negative health consequences associated with overnutrition, is desirable. The pattern of growth required to achieve this balance is unknown.

Based on the above, we performed a secondary analysis based on a published cohort study to explore the association between the early postnatal weight gain velocity and neurodevelopmental impairment at 24 months corrected age (CA) in the VLBWIs, thus providing more evidence in support of the association between the two variables and helping find a pattern of growth that facilitates optimal neurodevelopment yet minimizes negative health consequences associated with overnutrition.

## Participants and methods

### Data resource

This study uses secondary analysis of a previously published prospective cohort study (16). The data were retrieved from the “PLOS ONE” database (https://journals.plos.org/). Since the data uploader has waived all copyright, a secondary analysis without infringing on the authors’ rights when the data was used. (Data from: Chung-Ting Hsu, Chao-Huei Chen, Ming-Chih Lin, Teh-Ming Wang, Ya-Chi Hsu, 2017. Postdischarge body weight and neurodevelopmental outcomes among VLBWPIs in Taiwan: A nationwide cohort study. https://journals.plos.org/plosone/article?id=10.1371/journal.pone.0192574). The interesting independent variable in the present work is the weight gain velocity in different periods (from birth to 6 months CA, 6 to 12 months CA, and 12 to 24 months CA). The dependent variable is NDI (dichotomous variable: 0 = Non-NDI, 1 = NDI).

### Study population

The original study nonselectively and consecutively collected data on VLBWPIs (with a birth weight of less than 1500 g, and with a birth weight ≤1500 g and gestational age at birth less than 37 weeks) registered at the Premature Baby Foundation of Taiwan between 2007 and 2011 (16). All 21 hospitals located across the island of Taiwan participated in the data collection (17). Inclusion criteria were premature infants with a birth weight less than 1500 g, and exclusion criteria included (1) babies with advanced intraventricular hemorrhage (grade III and grade IV), (2) chromosome anomalies, (3) death before the end of follow-up, and (4) without BSID-II scores or complete follow-up data. The data were obtained from the Taiwan Premature Infant Developmental Collaborative Study Group, which was established by the Premature Baby Foundation of Taiwan. The local ethics committee of Taiwan Premature Infant Developmental Collaborative Study Group approved this original study (16). Informed consent was waived because that data were accessed anonymously in this study.

### Variables

#### Weight gain velocity from birth to 6 months CA, 6 to 12 months CA, and 12 to 24 months CA

As body weight gain is a key indicator of the degree of nutrition and catch-up growth of neonates, early postnatal growth in most cases is defined by weight growth (18, 19). There are many methods to calculate the weight gain velocity include grams/kilogram/day (g/kg/d), grams/day (g/d), and change in z scores (20). However, z score differences based on cross-sectional growth charts can be confused with distorted reference data, leading to a false reading of growth velocity of VLBWPIs (21). According to a systematic review, one of the frequently used methods to calculate the weight gain velocity was g/d (20). Considering the order of magnitude of uniform units and make the results easier to understand and to guide clinical practice, we defined the weight gain velocity of weight as g/d (W2 − W1)/(t2 − t1) in this study.

The first 6 months might be the key period of neurodevelopment in moderately preterm infants (8). The first 12 months of life are the most sensitive period of catch-up in weight and length for very preterm infants. It would be significant in determining later cognitive function (22). Therefore, we have chosen the time points above for data analysis.

#### Neurodevelopmental impairment

Our interesting outcome variable was NDI. The detailed process of measure of NDI is defined as any of the following conditions: Mental Developmental Index (MDI) score below 70, Psychomotor Developmental Index (PDI) below 70, cerebral palsy, visual impairment, or hearing impairment (16, 23). MDI and PDI scores were generated by BSID-II (24, 25). Cerebral palsy was defined as the presence of any of the following disorders: spastic tetraparesis, spastic hemiparesis, spastic diplegia, spastic dyskinesia, or hypotonia at 24 months CA by the neurologist. Visual impairment is defined as amblyopia or blindness in any eye at 24 months CA by an ophthalmologist. Hearing impairment is defined as more than 30 decibels (dB) hearing loss in any ear at 24 months CA by an otologist. All children who had visual or hearing impairments would be followed up by an ophthalmologist or otologist (16).

#### Covariates

Covariates were selected in our study according to the previous literature (26–28). Based on the above principles, therefore, the following variables were used as covariates: (1) continuous variables: gestation age and birth bodyweight; (2) categorical variables: gender, small for gestational age, intraventricular hemorrhage mild, persistent pulmonary hypertension of newborn, hemodynamic significant patent ductus arteriosus, necrotizing enterocolitis, chronic lung disease, respiratory distress syndrome, surfactant use, indomethacin use, and extrauterine growth retardation. Chronic lung disease was defined as needing respiratory support with oxygen or positive pressure ventilation at the postmenstrual age of 36 weeks. Hemodynamic significant patent ductus arteriosus was defined as needing medical intervention or surgical ligation (16). Small for gestational age was defined as a birth body weight below the 10th percentile of the standard fetal growth curve (29). Extrauterine growth retardation was defined as a weight below the 3rd percentile of the growth curve at discharge (30).

#### Follow-up procedure

The interval between each follow-up was at 6, 12, and 24 months CA. Monitoring indicators at each follow-up included body weight and neurological and psychomotor performance at 24 months CA. According to the selection criteria, the study initially collected 4,636 participants in this study; afterward, 1,093 participants were excluded due to chromosome anomalies (*n* = 24), severe intraventricular hemorrhage (grade III–grade IV) (*n* = 360), or death (*n* = 709). At 24 months CA, 1,752 infants were not followed up. Finally, a total of 1,791 participants were left for data analysis. Details of recruitment and follow-up were described in the original literature (16).

#### Statistical analysis

Continuous variables are expressed as mean (standard deviation) (Gaussian distribution) or median (min, max) (Skewed distribution), and categorical variables are given as frequencies and percentages. *χ*^2^ (categorical variables), Student’s *t*-test (normal distribution), or Man–Whitney *U* test (skewed distribution) were used to detect the differences among different NDI (binary variable). We used univariate and multivariate binary logistic regression models to test the connection between the weight gain velocity from birth to 6 months CA, 6 to 12 months CA, and 12 to 24 months CA and NDI with three distinct models, respectively. Model 1 is the nonadjusted model with no covariates adjusted. Model 2 is the minimally adjusted model with only sociodemographic variables adjusted [gender (male, female), gestational age at birth, birth body weight]. Model 3 is the fully adjusted model with covariates presented in Table 1 adjusted.

**Table 1:** Baseline characteristics of participants.

	Minimum	Maximum	Non-NDI mean + SD/*N* (%)	NDI mean + SD/*N* (%)	*P*-value	*P*-value^a^
N			1,263	528		
Gestational age, mean ± SD, weeks	22	36	29.55 ± 2.50	28.83 ± 2.97	<0.01	<0.01
Birth body weight, mean ± SD, g	422	1500	1,182.22 ± 229.81	1088.20 ± 260.50	<0.01	<0.01
Body weight at 6 months CA, mean ± SD, kg	3.80	11.70	7.23 ± 1.05	6.99 ± 1.20	<0.01	<0.01
Body weight at 12 months CA, mean ± SD, kg	3.90	14.00	8.91 ± 1.18	8.59 ± 1.29	<0.01	<0.01
Body weight at 24 months CA, mean ± SD, kg	6.70	18.50	11.46 ± 1.51	11.00 ± 1.68	<0.01	<0.01
Weight gain velocity from birth to 6 months CA, mean ± SD, g/d	9.72	39.93	23.90 ± 3.73	22.94 ± 4.38	<0.01	<0.01
Weight gain velocity from 6 months CA to 12 months CA, mean ± SD, g/d	−10.56	34.44	9.39 ± 3.66	8.92 ± 4.33	0.03	0.01
Weight gain velocity from 12 months CA to 24 months CA, mean ± SD, g/d	−3.33	21.64	7.08 ± 2.48	7.08 ± 2.48	0.01	<0.01
Gender, *n* (%)					<0.01	—
Male			620 (49.09%)	314 (59.47%)		
Female			643 (50.91%)	214 (40.53%)		
Small for gestational age, *n* (%)					0.45	—
No			829 (65.74%)	334 (63.86%)		
Yes			432 (34.26%)	189 (36.14%)		
Mild intraventricular hemorrhage, *n* (%)					<0.01	—
No			919 (74.41%)	345 (68.18%)		
Yes			316 (25.59%)	161 (31.82%)		
Pulmonary hypertension of newborn arteriosus, *n* (%)					0.02	—
No			1,238 (98.25%)	506 (96.38%)		
Yes			22 (1.75%)	19 (3.62%)		
Significant patent ductus persistent, *n* (%)					0.01	—
No			833 (67.56%)	323 (61.41%)		
Yes			400 (32.44%)	203 (38.59%)		
Necrotizing enterocolitis, *n* (%)					0.31	—
No			1,233 (97.78%)	510 (96.96%)		
Yes			28 (2.22%)	16 (3.04%)		
Chronic lung disease, *n* (%)					<0.01	—
No			1,033 (82.11%)	354 (68.74%)		
Yes			225 (17.89%)	161 (31.26%)		
Respiratory distress syndrome, *n* (%)					<0.01	—
No			240 (19.02%)	73 (13.83%)		
Yes			1,022 (80.98%)	455 (86.17%)		
Surfactant use, *n* (%)					<0.01	—
No			893 (70.70%)	293 (55.49%)		
Yes			370 (29.30%)	235 (44.51%)		
Indomethacin use, *n* (%)					<0.01	—
No			927 (73.40%)	351 (66.48%)		
Yes			336 (26.60%)	177 (33.52%)		
Extrauterine growth retardation at birth, *n* (%)					<0.01	—
No			710 (56.53%)	240 (46.07%)		
Yes			546 (43.47%)	281 (53.93%)		

CA, corrected age; NDI, neurodevelopmental impairment; MDI, Mental Developmental Index; PDI, Psychomotor Developmental Index.

^a^ *P* value: If it is a continuous variable, use Kruskal–Wallis rank sum test to obtain, if the count variable has a theoretical number <10, use Fisher's exact probability test to obtain.

To test the robustness of our results, we performed a sensitivity analysis. We converted the weight gain velocity in different periods into a categorical variable according to the tercile and calculated the *P* for trend in order to verify the results of the weight gain velocity as the continuous variable and to examine the possibility of nonlinearity.

To account for the nonlinear relationship between the weight gain velocity in different periods and NDI, we also used the generalized additive model and the smooth curve fitting (penalized spline method) to further explore the shape of their relations. In addition, a two-piece-wise binary logistic regression model was also used to explain the nonlinearity further.

All the analyses were performed with the statistical software packages R (http://www.R-project.org, the R Foundation) and EmpowerStats (http://www. empowerstats.com, X & Y Solutions, Inc., Boston, MA, United States). *P* values less than 0.05 (two-sided) were considered statistically significant.

## Results

### Baseline characteristics of participants

The authors presented baseline demographics and clinical characteristics of included participants in Table 1. The population (*n* = 1791) at baseline, of whom 52.15% % were male, had a mean gestational age of 29.55 ± (2.50) weeks. Compared with infants who got an NDI, those who are non-NDI have a higher weight gain velocity from birth to 6 months CA. However, there is no difference between the two groups in the weight gain velocity from 6 to 12 months CA and 12 to 24 months CA. In addition, infants who were not followed up had a greater prevalence of necrotizing enterocolitis, respiratory distress syndrome, and chronic lung disease, and had less surfactant, indomethacin use, and extrauterine growth retardation (weight < 3rd percentile at discharge). However, gender, gestational age at birth, birth body weight, small for gestational age, extrauterine growth retardation (weight < 10th percentile at discharge), mild intraventricular hemorrhage, persistent pulmonary hypertension of newborn, significant patent ductus arteriosus, etc., were proved to be similar among the 1,752 infants who were not followed up and the 1,791 that were left (16).

### The results of multivariate analyses using a binary logistic regression model

For the weight gain velocity from birth to 6 months CA, in the fully adjusted model, 1 g/d increase of weight gain velocity was related to 5% decrease in risk of NDI *[odds ratio (OR) = 0.95, 95% confidence interval (CI) 0.92–0.99, p < 0.01]*; 1 g/d increase of weight gain velocity from 6 to 12 months CA was related to 3% decrease in risk of NDI *(OR = 0.97, 95% CI 0.94, 1.00, p = 0.049);* and 1 g/d increase of weight gain velocity from 12 to 24 months CA was related to 5% decreases in risk of NDI *(OR = 0.95, 95% CI 0.90, 0.99, p = 0.02)*. All results are statistically significant (Table 2).

**Table 2:** Results of univariate and multivariate analysis.

	Nonadjusted model		Minimally adjusted model		Fully adjusted model	
	OR (95% CI)	*P*	OR (95% CI)	*P*	OR (95% CI)	*P*
Weight gain velocity from birth to 6 months CA	0.94 (0.91–0.97)	<0.01	0.95 (0.93–0.98)	<0.01	0.95 (0.92–0.99)	<0.01
Weight gain velocity from birth to 6 months CA (three groups)						
Low	1.0		1.0		1.0	
Medium	0.68 (0.53–0.88)	<0.01	0.75 (0.58–0.98)	0.04	0.81 (0.61–1.08)	0.15
High	0.70 (0.55–0.90)	<0.01	0.83 (0.62–1.10)	0.18	0.85 (0.63–1.16)	0.30
*P* for trend text	<0.01		0.18		0.30	
Weight gain velocity from 6 to 12 months CA	0.97 (0.94–1.00)	0.03	0.97 (0.94–0.99)	0.02	0.97 (0.94–1.00)	0.049
Weight gain velocity from 6 to 12 months CA (three groups)						
Low	1.0		1.0		1.0	
Medium	0.68 (0.52–0.88)	<0.01	0.69 (0.53–0.90)	0.01	0.69 (0.52–0.92)	0.01
High	0.74 (0.58–0.95)	0.02	0.74 (0.57–0.95)	0.02	0.76 (0.58–1.00)	0.05
*P* for trend text	0.02		0.02		0.06	
Weight gain velocity from 12 to 24 months CA	0.94 (0.90–0.99)	0.01	0.95 (0.91–0.99)	0.02	0.95 (0.90–0.99)	0.02
Weight gain velocity from 12 to 24 months CA (three groups)						
Low	1.0		1.0		1.0	
Medium	0.75 (0.58–0.97)	0.03	0.75 (0.58–0.98)	0.04	0.77 (0.58–1.01)	0.06
High	0.74 (0.57–0.96)	0.02	0.77 (0.59–1.00)	0.05	0.76 (0.58–1.01)	0.06
*P* for trend text	0.02		0.05		0.06	

CA, corrected age; OR, odds ratio; CI, confidence interval.

Nonadjusted model: covariates were not adjusted; minimally adjusted model: only gender, gestational age, birth body weight are adjusted for; fully adjusted model: all covariates presented in Table 1 (gender, gestational age, birth body weight, small for gestational age, mild intraventricular hemorrhage, significant patent ductus arteriosus, persistent pulmonary hypertension of newborn, necrotizing enterocolitis, chronic lung disease, respiratory distress syndrome, surfactant use, indomethacin use, and extrauterine growth retardation) are adjusted for.

To verify the robustness of our findings, a sensitivity analysis was performed. We first convert different periods of the weight gain velocity from continuous variables to categorical variables (according to tercile), then put these categorical variables back into the model. The results (Table 2) show that after the weight gain velocity from 6 to 12 and 12 to 24 months CA were transformed into categorical variables, the trend of the effect sizes in different groups is equidistant, but *P* for trend (*P = 0.30*) are inconsistent with the result (*P < 0.05*) when the weight gain velocity is a continuous variable, suggesting that the clarification of nonlinearity between the weight gain velocity on NDI is necessary.

### The nonlinearity addressed by the generalized additive model

**Figure 1:**
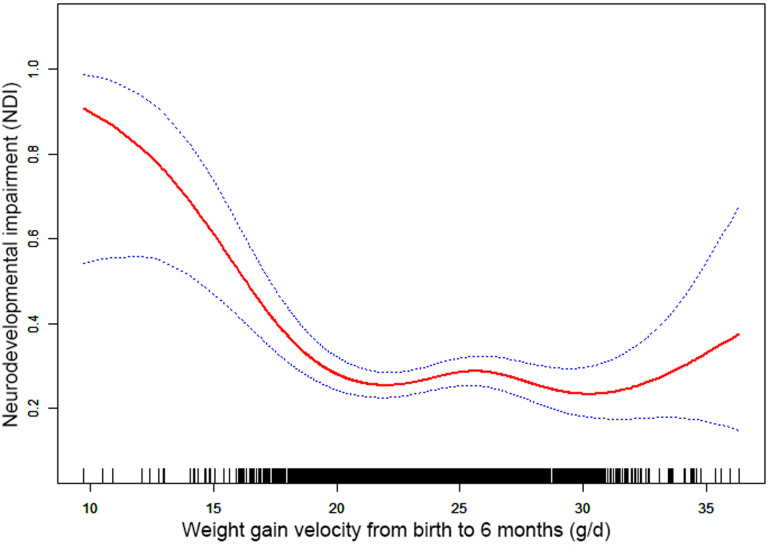
Nonlinearity of weight gain velocity from birth to 6 months CA on neurodevelopmental impairment.

**Figure 2:**
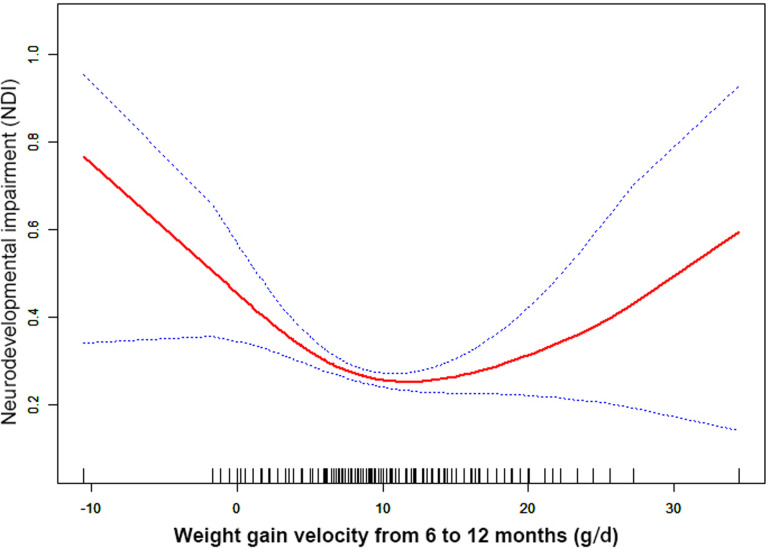
Nonlinearity of weight gain velocity from 6 to 12 months CA on neurodevelopmental impairment.

**Figure 3:**
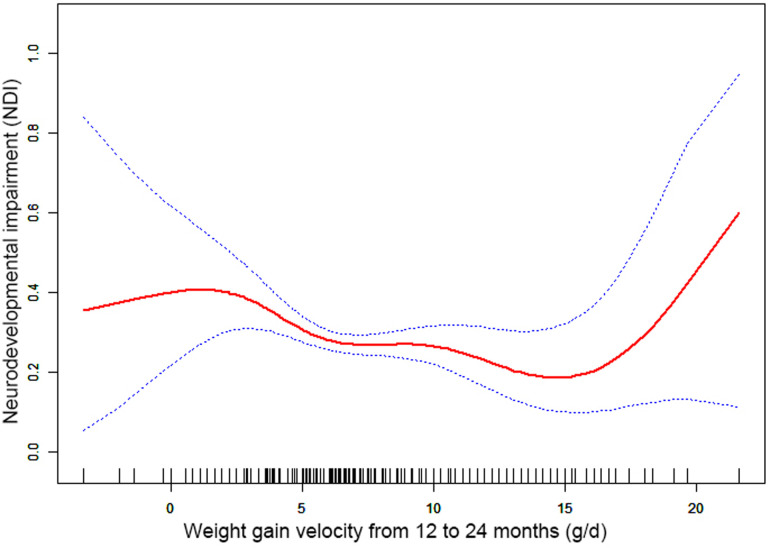
Nonlinearity of weight gain velocity from 12 to 24 months CA on neurodevelopmental impairment.

Through the generalized additive model and smooth curve fitting, we observed that from birth to 6 months CA and 6 to 12 months CA, the correlation between the weight gain velocity and NDI is nonlinear. However, there is no nonlinear relationship between the weight gain velocity from 12 to 24 months CA and NDI (Table 3). In our study, the *P* for log-likelihood ratio test of the relationship between NDI and both of the weight gain velocity from birth to 6 months CA and from 6 to 12 months CA was less than 0.01, so we used a two-piece-wise model to fitting the correlation between the weight gain velocity during this two periods and NDI. By recursive algorithm, the inflection points obtained first were 20.36 and 9.44 in those two sensitive periods, respectively. Then, the effect sizes and confidence interval on the left and right of the inflection point were calculated by a two-piece-wise binary logistic regression model. The results revealed that there are ceiling effects between the weight gain velocity from birth to 6 months CA and 6 to 12 months CA and NDI. For the weight gain velocity from birth to 6 months CA, on the left side of the inflection point, the rate is negatively associated with the risk of NDI *(inflection point 20.36,* <*inflection point: OR* *=* *0.75, 95% CI 0.67–0.84,* >*inflection point: OR* *=* *1.01, 95% CI 0.97–1.05)* (Figure 1); for the weight gain velocity from 6 to 12 months CA, the rate is negatively associated with the risk of NDI in the interval where the rate is less than 9.44 g/d *(inflection point 9.44,* <*inflection point: OR* *=* *0.89, 95% CI 0.84–0.94,* >*inflection point: OR* *=* *1.05, 95% CI 1.00–1.11)* (Figure 2), and for the weight gain velocity from 12 to 24 months CA, the rate is also negatively associated with the risk of NDI when the rate less than 16.00 g/d [inflection point 16.00, <inflection point: OR = 0.93, 95% CI 0.88–0.98, >inflection point: OR = 1.75, 95% CI 1.05 (0.96–3.08)] (Figure 3).

**Table 3:** Nonlinearity of weight gain velocity in different periods on neurodevelopmental impairment.

	Weight gain velocity from birth to 6 months CA		Weight gain velocity from 6 to 12 months CA		Weight gain velocity from 12 to 24 months CA	
	OR (95% CI)	*P*	OR (95% CI)	*P*	OR (95% CI)	*P*
Fitting model using binary logistic regression	0.95 (0.92–0.99)	<0.01	0.97 (0.94–1.00)	0.0493	0.95 (0.90–0.99)	0.02
Inflection point	20.36		9.44		16.00	
<Inflection point	0.75 (0.67–0.84)	<0.01	0.89 (0.84–0.94)	<0.01	0.93 (0.88–0.98)	<0.01
>Inflection point	1.01 (0.97–1.05)	0.63	1.05 (1.00–1.11)	0.05	1.75 (0.96–3.08)	0.05
*p* for log-likelihood ratio test	<0.01		<0.01		0.02	
95% CI of inflection point	18.89–21		8.34–9.99		—	

CA, corrected age; OR, odds ratio; CI, confidence interval.

The adjustment strategy was the same with fully adjusted model (gender, gestational age, birth body weight, small for gestational age, mild intraventricular hemorrhage, significant patent ductus arteriosus, persistent pulmonary hypertension of newborn, necrotizing enterocolitis, chronic lung disease, respiratory distress syndrome, surfactant use, indomethacin use, extrauterine growth retardation).

## Discussion

In this study, the results revealed that there were different relationships between the different periods of the weight gain velocity and neurodevelopmental outcomes in VLBWPIs. Surprisingly, we find it is not a simple linear relationship, but nonlinearity between early postnatal growth and NDI in the first year of CA. From birth to 6 months CA, a ceiling effect exists. If the rate is less than 20.36 g/d, the higher the weight gain velocity, the less risk of NDI. From 6 to 12 months CA, the higher the weight gain velocity, the less risk of NDI when the rate is less than 9.44 g/d. Moreover, we also found there is a nonlinear relationship between the weight gain velocity from 12 to 24 months CA and NDI, and the higher weight gain velocity is related with less risk of NDI when the rate is less than 16.00 g/d. The results did not appear to be fully compatible with other literature studies on this theme. Belfort et al. (31) conducted a study to identify the association between the linear slopes of weight growth from 1 week of age to term, term to 4 months, and 4–12 months, and the MDI and PDI scores at 18 months CA of 613 infants’ gestation age less than 33 weeks by linear regression analysis, finding that only the period from term to 4 months was the sensitive period of postnatal growth for preterm infants relative to neurodevelopment. There is no increase in indicators associated with MDI or PDI scores from 4 to 12 months and the faster weight gain, and linear growth from term to 4 months are associated with increased PDI scores at 18 months CA in preterm infants. Sammallahti et al. (32) identified weight growth between birth, 5 and 20 months CA, and 56 months to predicted neurocognitive abilities at 26 years of age, and the results showed that faster birth-to-5-months weight growth was the sensitive period associated with higher intelligence quotient (IQ), but no associations between neurocognitive after 5 months CA were identified. The above studies all found the linear relationship between weight growth and neurodevelopmental outcome among prematurity in the early stage after birth, but did not detect any associations between those two variables later in life. The widespread growth restriction in early postnatal might be the primary factor of those distinctions. Additionally, the first year of life, a sensitive period of human brain growth with the highest rate (33), is the key period for catch-up growth and later intelligence of VLBWPIs (34). The growth of very-low-weight preterm infants at discharge remains below the expected developmental level despite aggressive nutrition supplementation (35). Approximately one-third of preterm infants within the first 3 months after discharge would suffer a variety of feeding difficulties until the first year of their life (36, 37). Therefore, during the beginning months of postnatal, VLBWPIs need a higher growth velocity to achieve the body demand of catch-up growth. However, there might be a nonlinearity between both the weight gain velocity during the first year in postnatal and NDI, so those studies fail to detect it just by linear regression analysis. Nevertheless, there are still a lot of unknown fields about the mechanisms of relationships between the different periods of the weight gain velocity and neurodevelopmental outcomes in VLBWPIs that need to be explored.

Other studies stated that there might be a nonlinear association between early postnatal growth and neurodevelopmental outcome. Pylipow et al. revealed that an inverted J-shape curve existed between the postnatal growth rate in the first 4 postnatal months of infants with intrauterine growth restriction and later cognitive function (7). Taine et al. performed a systematic review of the relationship between early postnatal growth before age 3 years and neurodevelopmental outcome in children born moderately preterm or small for gestational age at term, and they found that few articles revealed a plateau for IQ with higher weight gain. It may suggest a possible ceiling effect (8). The possible reasons for this inconsistency may be related to the study population, study design, adjustment for covariates, especially the timing or tool of measurement (NICU hospitalization or postnatal; at gestational age or chronological age, different outcome indicators of neurodevelopmental impairment), or calculation (g/kg/d, g/d, cm/week, or change in z scores) of the weight gain velocity (20, 21, 38).

Furthermore, although the brain, corticospinal tract growth, and neurodevelopment in preterm infants after early postnatal energy- and protein-supplemented diet may be improved (39, 40), and malnutrition in a sensitive period during early neonatal life may lead to long-term neurodevelopment impairment (33, 41), but, conversely, rapid weight gain velocity among VLBWPIs promote higher long-term risk of overweight/obesity and other noncommunicable diseases, such as insulin resistance and metabolic syndrome, cardiovascular risk, adiposity, higher blood pressure, insulin resistance, dyslipidemia, and endothelial dysfunction (12, 13). The present study revealed that there are ceiling effects between the weight gain velocity from birth to 6 months CA and 6 to 24 months CA and NDI. For the weight gain velocity from birth to 6 months CA, 1 g/d increase of weight gain velocity is related to 25% decreases in risk of NDI when the rate is less than 20.36 g/d. For the weight gain velocity from 6 to 12 months CA, 1 g/d increase of weight gain velocity was related to 11% decreases in risk of NDI when the rate is less than 9.44 g/d. For the weight gain velocity from 12 to 24 months CA, 1 g/d increase of weight gain velocity was related to 7% decreases in risk of NDI the rate is less than 16.00 g/d. It means the neurodevelopmental benefits from a rapid weight gain velocity from birth to 24 months CA might be limited once the growth pace reaches an optimum level. Confirming the sensitive period of the relationship between weight gain velocity and neurodevelopment can help find a pattern of growth that facilitates optimal neurodevelopment, yet minimizes negative health consequences associated with overnutrition. According to these results of the study, we could keep the weight gain velocity of VLBWPIs close to those optimum levels in corresponding window period instead of gaining weight as fast as possible, as a result, to get enough the neurodevelopment benefits and simultaneously decrease the risk of long-term metabolic disorders. However, more evidence on the practical aspects for action should be explored deeply.

### Strengths of the study

Our study has some strengths listed as follows. A strength of our study is the large sample size that allows such analysis, whereas most prior studies were limited to small numbers. Compared with previous research, the research on the nonlinearity addressing is a significant improvement. It may propose a new insight into the association between those two variables and give some clue for further studies on this theme, and then give more evidence on the practical aspects for action finally. Additionally, this study is an observational study and therefore susceptible to potential confounding, so we used strict statistical adjustment to minimize residual confounders. We tested the robustness of the results through a series of sensitivity analyses (target independent variable transformation, subgroup analysis, log-likelihood ratio test, etc.) to ensure the reliability of the results.

### Limitations

Our research has the following shortcomings. First, our findings can be generalized to VLBWPIs only, and the relationship of the weight gain velocity on NDI may be different in babies with advanced intraventricular hemorrhage (grade III and grade IV) or chromosome anomalies. Second, as in all observational studies, even though known potential confounders factors were controlled for, there might have been still uncontrolled confounders. Third, it should be noticed that the causality between the weight gain velocity and NDI among VLBWPIs could not be addressed and the bidirectional interrelation may exist between these two variables. Further studies regarding identifying the mechanism of the relationship between weight gain velocity and NDI should be recommended.

## Conclusions

We explore the association between the weight gain velocity in different periods of early postnatal (from birth to 6 months CA, 6 to 12 months CA, and 12 to 24 months CA) and neurodevelopmental impairment among VLBWPIs. The results show there is not simple linear relationship between the weight gain velocity form birth to 12 months CA and NDI among VLBWPIs. Ceiling effects was identified between less NDI and higher weight gain velocity in this group during birth to 6 months CA and 6 to 12 months CA. For the weight gain velocity from birth to 6 months CA, 1 g/d increase of weight gain velocity is related to 25% decrease in risk of NDI. For the weight gain velocity from 6 to 12 months CA, 1 g/d increase of weight gain velocity was related to 11% decrease in risk of NDI. For the weight gain velocity from 12 to 24 months CA, 1 g/d increase of weight gain velocity was related to 7% decrease in risk of NDI; the rate is less than 16.00 g/d. Assessing causality between the NDI and the weight gain velocity in an observational study is very difficult. But at the minimum, the exposure occurrence before the outcome and potential confounding variables were assessed and controlled. The results reveal that the neurodevelopmental benefits from a rapid weight gain velocity from birth to 12 months CA might be limited once the growth pace reaches an optimum level, while it may not be limited with the weight gain velocity after 12 months CA. It provides new insight into the association between the early postnatal the weight gain velocity and neurodevelopmental outcome. Further studies regarding identifying the mechanism of the relationship between weight gain velocity and NDI should be recommended to help us find the pattern of manage nutrition and weight gain curves that facilitate optimal neurodevelopment, yet minimizes negative health consequences associated with overnutrition further.

## Data Availability

The original contributions presented in the study are included in the article, further inquiries can be directed to the corresponding author.
